# Dual-pathway processing of AI-generated Chinese ink painting: evidence from eye-tracking, EEG, and artistic expertise

**DOI:** 10.3389/fpsyg.2026.1966943

**Published:** 2026-09-29

**Authors:** Qiang Qin, Jing Chai, Wanni Zhong

**Affiliations:** 1 Academy of Arts & Design, Tsinghua University, Beijing, China; 2 College of Fine Arts, Capital Normal University, Beijing, China; 3 College of Fine Arts, Hongik University, Seoul, Republic of Korea

**Keywords:** algorithmic artistic conception, Chinese ink painting, expressive expansion, neuroaesthetics, transmedia convergence

## Abstract

AI-driven generative art is changing the scope of artistic creation, but the principles behind its aesthetics and the perception of its outcomes remain relatively unexplored, especially concerning culturally specific art forms. The present study investigates how viewers cognitively, affectively, and evaluatively process AI-generated Chinese ink paintings, and whether artistic expertise moderates these responses. We introduce the concept of expressive expansion as a viewer-perceived psychological effect — the tendency for AI-generated ink works to elicit higher ratings of expressiveness and dynamic tension than traditional counterparts — and examine its neural and behavioral correlates using a multimodal paradigm. Stimuli generated using a pre-trained Stable Diffusion architecture were assessed according to three aesthetic metrics along with traditional and digital non-AI artworks by 120 respondents equally split into professional artists and non-artists. Multidimensional measurement based on eye-tracking, semantic differential rating, and EEG showed that, under blind viewing conditions, AI-generated stimuli yielded significantly greater scan-path entropy and beta band suppression than traditional and digital non-AI works, suggesting that they engaged broader attentional exploration and increased attentional engagement as indexed by implicit behavioral and neural measures. Professional and non-professional evaluators agreed on expressiveness evaluation scores but differed considerably in their cultural authenticity perception, suggesting a dissociation between aesthetic appeal and cultural validity. These findings are consistent with a dual-pathway interpretive framework in which perceptual-affective and cultural-evaluative responses can dissociate as a function of artistic expertise — an account advanced as consistent with the observed dissociation rather than as a mechanism established by the present data — and contribute to the emerging empirical literature on audience responses to AI-generated art in culturally specific domains. All conclusions are restricted to the selected stimulus sets under the present experimental conditions. The source code, LoRA model weights, and experimental datasets will be made publicly available at: https://github.com/15100376058/ink-painting-ai-expressive-expansion (DOI: 10.5281/zenodo.20540420).

## 1 Introduction

The fast development of generative AI technology has irrevocably changed the dynamics of creativity, affecting both the processes of image creation and interpretation (Epstein et al., 2023). One of the most interesting cultural traditions that has found itself in the focus of this technological evolution is the practice of ink painting from China, which carries centuries of aesthetic philosophy embedded in its techniques of managing empty space, brushstroke application, and tonal variation. The issue of what will happen to the tradition of ink painting once it is disassembled by a machine is thus far more than an aesthetic problem; it becomes a question of authorship and artistic meaning.

The psychological study of aesthetic experience has matured substantially over the past two decades, evolving from early psychophysical approaches into the interdisciplinary field of empirical aesthetics and its neuroscientific extension, neuroaesthetics (Chatterjee and Vartanian, 2014; Vessel et al., 2012). Contemporary frameworks treat aesthetic response as a multidimensional construct encompassing perceptual fluency, affective valence, cognitive engagement, and evaluative judgment, each of which can be measured through behavioral and neural methods (Chatterjee and Vartanian, 2014). A particularly influential development has been the application of electroencephalography (EEG) to aesthetic experience: frontal alpha asymmetry (FAA) has been established as a reliable index of approach-oriented affect during artwork viewing, while beta band suppression has been associated with states of deep perceptual engagement and aesthetic absorption (Welter and Lotte, 2024; Shemyakina et al., 2024). These neural indices offer a principled bridge between the phenomenology of aesthetic experience and its measurable neural correlates, making them well-suited for investigating audience responses to novel stimulus categories such as AI-generated art — a domain in which traditional self-report measures may be insufficient to capture the full range of implicit and explicit response components.

A central concern in empirical aesthetics is how artistic expertise modulates aesthetic perception and evaluation. Converging evidence indicates that expertise does not produce uniform enhancement of aesthetic response; rather, it selectively amplifies responses on culturally and cognitively demanding dimensions while leaving more immediate affective reactions largely intact. Art-trained viewers reliably give higher ratings on beauty and preference dimensions than novices, but the two groups show comparable emotional arousal and valence responses to the same stimuli — a dissociation consistent with the view that expertise primarily expands the cognitive-evaluative repertoire available during viewing rather than heightening basic affective reactivity (Vessel and Rubin, 2010; Van Paasschen et al., 2015). Expertise further modulates the interaction between style and context: experts show greater differentiation between established and unestablished works and between abstract and representational styles, suggesting that specialized schema acquisition reshapes the criteria by which aesthetic value is assigned (Li, 2025). In the domain of ink painting specifically, where canonical quality criteria include technically demanding dimensions such as brushstroke intentionality, compositional harmony, and cultural codification, expertise effects are expected to be particularly pronounced on culturally grounded evaluative dimensions, while perceptually salient features such as dynamic tension and spatial novelty may remain more equally accessible across expertise levels.

Eye-tracking methodology has become a cornerstone of empirical aesthetics research, enabling direct measurement of the attentional processes that unfold during artwork viewing. A robust finding is that gaze behavior during art viewing is systematically structured rather than random: viewers allocate fixations preferentially to regions of high saliency, semantic content, and compositional weight, with scan-path characteristics varying as a function of both stimulus properties and viewer expertise (Casteau and Smith, 2024). Of particular relevance to the present study is the relationship between visual ambiguity and scan-path entropy: more ambiguous or compositionally complex stimuli elicit more spatially dispersed and exploratory gaze trajectories, reflecting sustained attentional search in the service of meaning construction (Cunningham et al., 2025). Recent integrative work has further demonstrated that scan-path dynamics and concurrent EEG oscillatory patterns jointly index aesthetic connoisseurship, with expert viewers exhibiting systematically distinct oculomotor–neural coupling compared to novices during artwork viewing (Cheron and De Maere, 2025). These findings establish scan-path entropy as a theoretically grounded behavioral index of the depth of perceptual engagement, motivating its use in the present study as an implicit measure of expressive expansion effects. The present study builds directly on this multimodal tradition, in which eye-tracking, EEG, and combined oculomotor–neural approaches (Welter and Lotte, 2024; Shemyakina et al., 2024; Cheron and De Maere, 2025; Arioli et al., 2025) jointly inform the neural and behavioral correlates of aesthetic processing, positioning the present design within an established body of neuroaesthetic measurement rather than an isolated paradigm.

A growing body of research has examined how knowledge of authorship — specifically, whether a work is known to be human- or AI-created — modulates aesthetic evaluation and authenticity judgment. The evidence is complex and sometimes counterintuitive. When authorship information is withheld, viewers show no systematic preference for human over AI-generated artworks and in some cases prefer AI-generated images under blind viewing conditions (Van Hees et al., 2025). However, when AI authorship is disclosed, aesthetic ratings typically decline and perceived authenticity drops substantially, suggesting that the devaluation of AI art is driven primarily by attribution effects rather than perceptual inferiority (Di Dio et al., 2023; Messingschlager and Appel, 2025). The mechanism underlying this devaluation appears to involve the perceived absence of human intentionality and embodied creative process: viewers implicitly expect artworks to carry the trace of a deliberate, embodied agent, and the revelation of algorithmic authorship disrupts this expectation (Messingschlager and Appel, 2025). In culturally specific art forms such as Chinese ink painting, where the embodied dimension of brushstroke execution carries particular aesthetic and philosophical weight, this attribution effect may be especially pronounced among viewers with deep cultural expertise — producing a dissociation in which high aesthetic appeal coexists with low cultural-legitimacy judgment specifically when professional viewers assess AI-generated works.

Theoretical accounts of aesthetic evaluation have increasingly converged on dual-process frameworks that distinguish between fast, implicit, perceptually driven responses and slower, deliberative, culturally mediated evaluations (Leder et al., 2004; Pelowski et al., 2017). Within such frameworks, the initial encounter with an artwork is characterized by rapid affective registration — sensitivity to visual complexity, compositional balance, and tonal structure — that occurs largely independently of semantic or cultural knowledge. This fast route feeds into a second, slower evaluation process in which the viewer applies culturally acquired criteria, domain-specific schema, and biographical knowledge to form explicit judgments of quality, authenticity, and meaning (Leder et al., 2004). The dissociation between implicit and explicit processing routes predicts that groups differing in cultural expertise should diverge most strongly on dimensions that recruit the slower evaluative route — precisely the culturally grounded dimensions of authenticity and tradition adherence — while converging on dimensions driven primarily by perceptual salience. This theoretical architecture maps directly onto the present study’s design, in which eye-tracking and EEG index the implicit perceptual-affective route, while semantic differential ratings capture the explicit evaluative route, and expertise group membership serves as the primary moderator of the divergence between them.

From a technical standpoint, generating plausible Chinese ink paintings presents challenges that conventional style transfer methods cannot adequately address, owing to the asymmetric brushstroke structure, tonal diffusion behavior, and culturally specific compositional conventions of the ink painting tradition (Wang et al., 2023; Gao and Zhang, 2025; Wang et al., 2026). Recent advances in latent diffusion models, attention-based architectures, and lightweight style transfer networks have substantially improved the fidelity of AI-generated ink paintings (Chen and Zhang, 2024; Lyu et al., 2025; Rombach et al., 2022; Tan, 2022; Tang, 2022; Zhang et al., 2024), enabling the construction of stimulus sets that are visually credible as ink painting exemplars while systematically varying along theoretically motivated aesthetic dimensions. These technical developments provide the generative infrastructure underlying the present study’s stimulus construction, the details of which are described in Section 3.1.

Beyond technical considerations, AI-generated ink paintings raise culturally specific questions about the transmission and transformation of a tradition in which the embodied relationship between artist, brush, ink, and paper carries deep aesthetic and philosophical significance (Worden, 2023; Xu et al., 2025; Li et al., 2025; Yang et al., 2025). The digital museum context has increasingly foregrounded these tensions, as generative technologies are deployed not merely to replicate heritage artifacts but to activate and reinterpret their meanings in ways that challenge established criteria of authenticity and authorship (Xu et al., 2025). Chinese ink painting is a particularly salient stimulus domain for investigating these tensions empirically: its aesthetic philosophy explicitly valorizes the productive role of accident and spontaneity (‘contingency/accident’, 偶然性) — a core Chinese ink painting concept referring to the generative role of uncontrolled, spontaneous ink behavior, the functional deployment of empty space (‘leaving blank’, 留白), and the transmission of vital force through gestural energy (‘breath-rhythm’, 气韵) — dimensions that map onto psychologically meaningful constructs of visual ambiguity, compositional balance, and perceived dynamism that are amenable to behavioral and neural measurement (Arioli et al., 2025).

These theoretical tensions converge on a set of fundamentally psychological questions that remain empirically underspecified. When viewers encounter an AI-generated ink painting, what cognitive and affective processes are engaged? Does the visual ambiguity and dynamic tension characteristic of algorithmically generated compositions activate broader attentional exploration, as indexed by gaze behavior? Does exposure elicit approach-oriented affect comparable to — or exceeding — that induced by traditional works, as measurable through frontal neural asymmetry? And critically, does the answer depend on who is looking: do art professionals, whose perceptual schemas are shaped by years of engagement with the embodied conventions of ink painting, respond differently from non-professionals whose aesthetic responses are less constrained by cultural-evaluative frameworks? Addressing these questions requires repositioning the problem of AI-generated ink art from an art-theoretical debate about authenticity and tradition into an empirical investigation of audience perception, aesthetic judgment, and expertise-dependent processing.

Against this backdrop, the present study addresses the following central research question: How do viewers cognitively, affectively, and evaluatively process AI-generated Chinese ink paintings, and how does artistic expertise moderate these responses? To investigate this question, we adopt a multimodal experimental paradigm combining eye-tracking, semantic differential scaling, and EEG-based affective measurement, applied to a stimulus set comprising traditional ink originals, AI-generated ink works, and digitally executed non-AI ink paintings viewed by professional artists and non-professionals.

We operationalize the construct of expressive expansion not as an inherent property of AI-generated images, but as a viewer-perceived psychological effect: the pattern whereby AI-generated ink works elicit systematically distinct perceptual and affective responses relative to tradition-bound originals. Within the dual-pathway framework elaborated in Section 2, which integrates established findings on expertise-dependent aesthetic processing (Vessel and Rubin, 2010; Van Paasschen et al., 2015; Li, 2025), the role of visual ambiguity in gaze behavior (Casteau and Smith, 2024; Cunningham et al., 2025; Cheron and De Maere, 2025), and attribution effects in AI art evaluation (Van Hees et al., 2025; Di Dio et al., 2023; Messingschlager and Appel, 2025), we derive the following *a priori* hypotheses:

*H1*: AI-generated ink works will receive significantly higher viewer ratings of expressiveness and innovativeness than traditional originals and digital non-AI works, reflecting perceptual-affective route engagement.

*H2*: AI-generated ink works will elicit significantly higher scan-path entropy than comparison conditions, indicating broader attentional exploration consistent with expressive expansion activating meaning-search processes.

*H3*: AI-generated ink works will produce stronger EEG beta suppression and higher frontal alpha asymmetry scores than comparison conditions, tentatively interpreted as consistent with, rather than uniquely indicative of, deeper aesthetic immersion and approach-oriented affect, given that these oscillatory indices admit multiple functional interpretations.

*H4*: Expertise will significantly moderate responses on culturally grounded dimensions (cultural authenticity, cultural resonance) but not on perceptually driven dimensions (expressiveness, innovativeness), such that professionals rate AI-generated works lower on authenticity than non-professionals.

*H5*: Across participants, scan-path entropy will positively correlate with expressiveness ratings, and beta suppression magnitude will positively correlate with overall aesthetic evaluation scores, linking implicit behavioral and neural indices to explicit aesthetic judgment.

*H6*: The divergence between expressiveness ratings and cultural authenticity ratings for AI-generated works — operationalized as the dissociation — will be significantly larger in the professional group than in the non-professional group.

These hypotheses are grounded in converging empirical literatures. H1 and H4 draw on evidence that artistic expertise selectively amplifies responses on culturally and cognitively demanding dimensions while leaving immediate perceptual-affective reactions largely intact (Vessel and Rubin, 2010; Van Paasschen et al., 2015; Li, 2025). H2 builds on findings that visually ambiguous and compositionally complex stimuli elicit more spatially dispersed and exploratory gaze trajectories (Casteau and Smith, 2024; Cunningham et al., 2025; Cheron and De Maere, 2025). H3 is based on neuroaesthetic evidence linking beta-band suppression and frontal alpha asymmetry to aesthetic immersion and approach-oriented affect (Welter and Lotte, 2024; Shemyakina et al., 2024). H4 and H6 reflect attribution research showing that disclosure of AI authorship disproportionately lowers perceived authenticity, an effect expected to be strongest among viewers with deep cultural expertise (Van Hees et al., 2025; Di Dio et al., 2023; Messingschlager and Appel, 2025). H5 follows from the dual-process architecture of aesthetic evaluation, in which implicit indices and explicit judgments are hypothesized to be correlated when they draw on the same processing route (Leder et al., 2004; Pelowski et al., 2017).

## 2 Theoretical framework

The theoretical framework of the present study operates at two complementary levels. At the cultural-descriptive level, a three-stage transmedia convergence model situates AI-generated ink painting within the broader process of tradition transformation. At the psychological-explanatory level, a dual-pathway perceptual model provides an interpretive framework for how viewers process AI-generated works and explains how artistic expertise moderates these responses. The transmedia convergence model provides cultural context; the dual-pathway model constitutes the primary explanatory framework of the study.

The dual-pathway model, illustrated in Figure 1, proposes that audience responses to AI-generated ink paintings are organized along two functionally distinct processing routes that can dissociate from one another, particularly as a function of viewer expertise. The first is the perceptual-affective route, driven by bottom-up stimulus properties and operating largely independently of cultural knowledge. The characteristic visual properties of AI-generated ink paintings — heightened spatial ambiguity, dynamic compositional tension, and tonal complexity unconstrained by physical medium — activate attentional exploration and approach-oriented affect that are registered implicitly through gaze behavior and frontal neural responses. Because this route is primarily stimulus-driven, its outputs are predicted to be largely invariant across expertise groups: both professionals and non-professionals are expected to register the heightened expressiveness and innovativeness of AI-generated works at the perceptual level. The second is the cultural-evaluative route, driven by top-down application of culturally acquired schema through deliberative, expertise-dependent processing. Viewers with deep familiarity with the embodied conventions of ink painting apply tradition-based criteria when judging the authenticity and cultural legitimacy of a work, and because AI-generated paintings lack the embodied generative process that grounds these criteria, expert viewers are expected to assign significantly lower cultural authenticity ratings than non-professionals. The dissociation between these two routes — high perceptual-affective engagement coexisting with low cultural-evaluative endorsement — constitutes what we term a dissociation, operationalized as the divergence between expressiveness and cultural authenticity ratings for AI-generated stimuli specifically within the professional group. We emphasize that the dual-pathway model is advanced here as an interpretive framework consistent with the observed dissociating pattern of condition and expertise effects, rather than as a mechanism established by the present data, particularly because H5 — the direct test linking implicit and explicit measures — was not supported.

**Figure 1:**
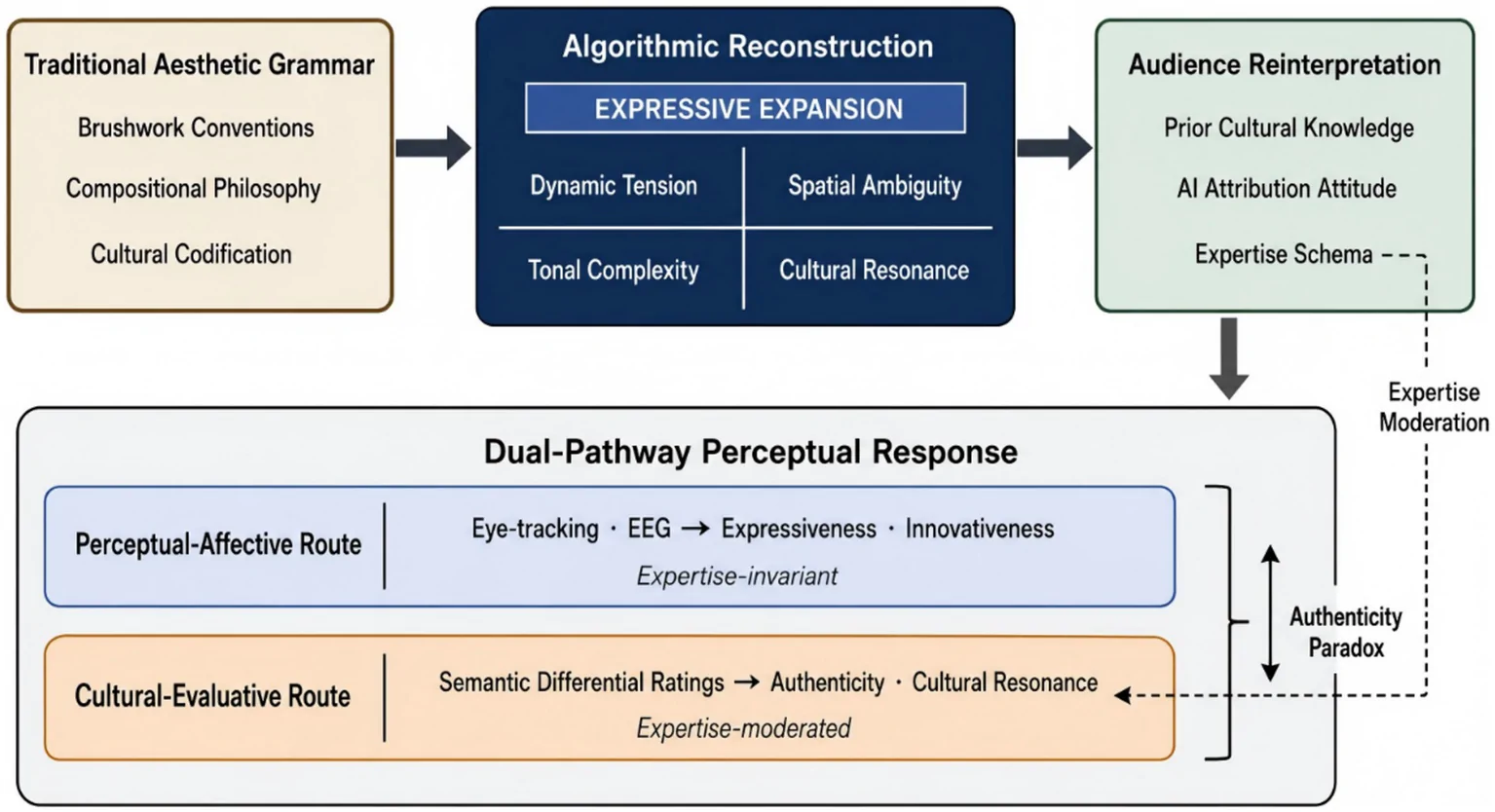
Dual-pathway perceptual model and three-stage transmedia convergence framework.

As cultural backdrop, the three-stage transmedia convergence model characterizes the broader process through which ink painting tradition interfaces with algorithmic generation and audience reception, as illustrated in Figure 1. The first stage, traditional aesthetic grammar, encompasses the visual philosophy and technical conventions — brushwork, negative space, and compositional rhythm — that constitute the cultural input to the generative process. The second stage, algorithmic reconstruction, represents the transformation of this grammar through latent diffusion modeling, wherein expressive expansion occurs: the generative system samples configurations in latent space that are unconstrained by physical medium and may occupy aesthetic territories inaccessible through controlled human brushwork. The third stage, audience reinterpretation, encompasses the full range of psychological processes described by the dual-pathway model, as modulated by prior cultural knowledge, attitudes toward AI, and expertise schema.

Within this integrated framework, expressive expansion is defined not as an inherent property of the generative system but as a viewer-perceived psychological effect — the tendency of AI-generated ink works to elicit higher ratings of expressiveness, dynamic tension, and spatial ambiguity than tradition-bound originals under matched viewing conditions, as registered through the perceptual-affective route. This dissociation, by contrast, emerges at the cultural-evaluative route and is specific to viewers with sufficient expertise to apply tradition-based legitimacy criteria. Together, these two constructs describe the two characteristic response patterns observed in the present study — perceptual-affective enhancement and cultural-evaluative attenuation — which, taken together, characterize how audiences respond psychologically to AI-generated ink art under the present experimental conditions.

Conceptual boundaries. Expressive expansion should be distinguished from several adjacent constructs. Visual novelty refers to the degree to which a stimulus deviates from familiar prototypes; perceived creativity denotes an explicit attribution of generative skill or originality; and aesthetic evaluation encompasses the overall valence and quality judgment of a work. Expressive expansion differs from each: it denotes a viewer-perceived intensification of expressive, dynamic, and ambiguous qualities — a perceptual-affective response pattern rather than a category judgment or an overall evaluative verdict. Operationally, it is indexed by elevated expressiveness and innovativeness ratings together with the implicit behavioral and neural measures described in Section 3, and it is conceptually anchored in the ink-painting aesthetic values of contingency (偶然性), breath-rhythm (气韵), and leaving-blank (留白) rather than in generic novelty or creativity.

## 3 Research design and methods

### 3.1 Construction of the AI ink painting generation system

The generative backbone of this study is a Stable Diffusion latent diffusion model selectively fine-tuned via Low-Rank Adaptation (LoRA) on a curated corpus of classical and contemporary Chinese ink paintings. The LoRA adaptation introduces trainable low-rank decomposition matrices into the attention layers of the UNet, such that the weight update for any given layer is expressed as:


$$W^{\prime }=W+\text{αrBA}$$

The LoRA-adapted model is then trained with the standard diffusion denoising objective:


$$L=E_{x_{0},\epsilon ,t}[{\left\|\epsilon -\epsilon_{\theta }(X_{t},t,c)\right\|}^{2}]$$

where x_0 is the clean image, ϵ~N(0,I) is the added noise, x_t is the noisy image at timestep t, c is the text conditioning signal, and $\epsilon_{\theta }$ is the UNet noise predictor parameterized by the adapted weights.

The central methodological contribution of the generation system lies in the construction of an ink painting semantic vector space that enables quantitative scoring of generated images on three culturally specific aesthetic dimensions grounded in classical Chinese aesthetic theory. Ink resonance (墨韵) captures the degree to which a generated image embodies the characteristic tonal gradation behavior of ink on xuan paper, including diffusion halos, dry-brush striations, and tonal continuity. Negative space (留白) indexes the functional deployment of unpainted regions as compositionally active elements, operationalized as a spatial entropy measure:


$$H=-\sum_{i}p(d_{i})\log p(d_{i})$$

computed over the probability distribution of figure-ground boundary distances, which captures whether empty regions are compositionally active in the manner prescribed by the classical principle of ‘leaving blank’ (留白) or merely incidental. Breath-rhythm (气韵) refers to the perceived dynamism and internal movement of the composition as a whole, operationalized through a directional energy field computed over the gradient magnitude map of the generated image:


$$E=\frac{1}{N}\sum_{i}\omega (x_{i})|\nabla I(x_{i})|$$

where $\nabla I(x_{i})$ is the image gradient at pixel x_i, $\omega (x_{i})$ is a spatial weighting function that down-weights peripheral regions consistent with traditional compositional focus conventions, and *N* is the total number of pixels.

Each dimension is scored using contrastive CLIP-based text anchors. The CLIP image encoder (ViT-L/14, output dimensionality 768) extracts image embeddings projected into a 128-dimensional aesthetic subspace via PCA fitted on an expert-annotated reference set. For each aesthetic dimension *d*, a positive anchor prompt *P^+^* and a negative anchor prompt *P^−^* are defined, and the dimension score for image *I* is computed as the cosine similarity differential:


$$S_{d}(I)=\cos(E_{I}(I),E_{T}(P_{d}^{+}))-\cos(E_{I}(I),E_{T}(P_{d}^{-}))$$

where *E_I_* denotes the CLIP image encoder and *E_T_* denotes the CLIP text encoder. Representative anchor prompt pairs for each dimension are provided in Table 1. A composite aesthetic score is then computed as the weighted linear combination:


$$S=\alpha S_{\mathrm{ink}}+\beta S_{\text{space}}+\gamma S_{\text{breath}}$$

**Table 1:** Contrastive text anchor prompt pairs for the three culturally specific aesthetic dimensions used in CLIP-based semantic vector space construction.

Dimension	Positive anchor prompt	Negative anchor prompt
Ink Resonance (墨韵)	ink wash with tonal gradation, diffusion halos, dry-brush texture on xuan paper	flat digital illustration with uniform color and hard edges
Negative Space (留白)	Chinese ink painting with vast empty white space, sparse compositional elements	densely packed composition with no unpainted regions
Breath-Rhythm (气韵)	dynamic ink composition with flowing directional energy, rhythmic spatial tension	static symmetrical composition with uniform weight distribution

Chinese terms: Ink Resonance (墨韵, mò yùn)—“ink resonance,” the tonal behavior and aesthetic vitality of ink wash; Leaving Blank (留白, liú bái)—“leaving blank,” the deliberate use of unpainted empty space as a compositional element; Breath-Rhythm / Spirit-Resonance (气韵, qì yùn)—“breath-rhythm” or “spirit-resonance,” the flowing vital energy and rhythmic movement in a work. These terms are retained in their original Chinese form because they are the canonical art-historical designations that lack precise single-word English equivalents.

where weights α, β, and γ were determined through ordinary least squares (OLS) regression against consensus ratings provided by ten expert ink painting practitioners on a held-out set of 60 generated images, yielding α = 0.41, β = 0.35, γ = 0.24 with an adjusted R^2^ = 0.81. Variance inflation factors for all three predictors were below 2.1, confirming the absence of multicollinearity among the aesthetic dimensions. The ink resonance dimension received the highest weight, consistent with expert consensus that tonal behavior constitutes the primary discriminating feature of authentic ink painting quality.

The generation pipeline produced stimuli by sampling from the fine-tuned model across three conditioning regimes corresponding to high, medium, and low values of the composite aesthetic score, yielding a stimulus space that varied systematically across the expressive expansion dimensions defined in the theoretical framework. In total, 300 images were generated before screening. All generated images were post-screened by two independent ink painting experts to exclude outputs containing obvious artifacts (e.g., anatomical distortions, text-like artifacts, and degraded ink textures); 240 images were excluded at this stage, and the final retained set comprised 60 images distributed evenly across the three aesthetic score tiers. From this retained set, the 20 experimental AI-generated images were selected to maximize balanced coverage of the three score tiers (6–7 images per tier) while approximating the genre and compositional-orientation distribution of the traditional and digital non-AI conditions, so that the three conditions did not differ systematically in genre composition. The two experts who performed the artifact screening were not involved in constructing the aesthetic scoring system, and none of the experts who participated in stimulus screening or scoring took part in the later stimulus validation ratings described in Section 3.2. All code, model weights, and configuration files are openly available at https://github.com/15100376058/ink-painting-ai-expressive-expansion (DOI: 10.5281/zenodo.20540420).

### 3.2 Stimulus material design

The stimulus set comprised 60 images organized into three matched groups of 20, designed to isolate the perceptual effects attributable specifically to AI generation rather than to digital mediation or stylistic novelty more broadly. The same genre, dominant compositional orientation, and quality-threshold criteria were applied symmetrically across the three conditions. The first group, traditional ink originals, consisted of high-resolution digitized reproductions of authenticated classical and modern ink paintings drawn from an initial candidate pool of 80 digitized works, from which images of insufficient scan quality or ambiguous provenance were excluded; the final 20 works were selected to maximize canonical representativeness and genre coverage (landscape, figure, bird-and-flower). The second group, AI-generated ink works, was drawn directly from the output of the fine-tuned generation system described in Section 3.1, with images selected to span the three aesthetic score tiers defined by the composite score S and screened by expert review to exclude technical artifacts. The third group, digital ink works, comprised non-AI digital paintings executed in ink-painting style using professional illustration software by trained human artists. Artists for this group were recruited through art academies and professional illustration platforms, and were required to meet the following inclusion criteria: a minimum of five years of formal training or professional practice in Chinese ink painting, documented proficiency in digital illustration tools (Procreate or Adobe Fresco), and no prior involvement in AI-assisted art production. A total of eight artists were commissioned to produce the 20 digital works, with each artist contributing two to three pieces to ensure stylistic diversity within the group. To independently verify the quality and stylistic authenticity of the digital works, all 20 images were rated by a panel of three ink painting experts who were blind to the study hypotheses and who evaluated each work on technical proficiency, stylistic fidelity to the ink painting tradition, and overall compositional quality using a seven-point scale. Only images receiving a mean expert rating of 4.5 or above were retained, and three initially produced works that fell below this threshold were replaced through additional commissioned production. This verification procedure ensured that the digital non-AI condition represented a credible and high-quality baseline against which the effects of algorithmic generation could be meaningfully assessed. None of the three validation experts for the digital works had been involved in constructing the aesthetic scoring system or in screening the AI-generated stimuli; all three were naive to the study hypotheses.

Across all three groups, images were matched as closely as possible on genre, dominant compositional orientation, and overall luminance distribution, with mean luminance constrained to fall within one standard deviation of the cross-group mean $\bar{L}\pm \sigma_{L}$ to prevent low-level tonal differences from confounding aesthetic judgments. All images were standardized to 1024 ×1024 pixels and presented on a calibrated display under controlled luminance conditions. Authorship information was withheld from participants throughout the experiment to prevent attribution effects from contaminating perceptual and affective responses, consistent with the implicit measurement logic of the dual-pathway framework outlined in Section 2.

To ensure that differences in observers’ responses across the three stimulus conditions could be attributed to their generative origin rather than to incidental variation in low-level visual properties, all images were subjected to a systematic physical matching procedure prior to experimental use. Six image-level indices were computed for every stimulus using standard computational pipelines: (1) mean luminance (mean pixel intensity in CIE L* space); (2) global contrast (root-mean-square contrast, defined as the standard deviation of pixel luminance values normalized to the range [0, 1]); (3) visual complexity (edge density, defined as the proportion of pixels classified as edges by a Canny edge detector with fixed threshold parameters applied uniformly across all images); (4) color saturation (mean saturation in HSV space); (5) hue diversity (standard deviation of hue values in HSV space); and (6) spatial-frequency distribution (spectral slope of the radially averaged power spectrum). One-way ANOVAs conducted separately for each of the three primary low-level indices confirmed that the three stimulus groups did not differ significantly on any of the three measures: mean luminance, *F* (2, 57) = 1.43, *p* = 0.248, η^2^p = 0.048; RMS contrast, *F* (2, 57) = 1.87, *p* = 0.163, η^2^p = 0.062; edge density, *F* (2, 57) = 2.11, *p* = 0.131, η^2^p = 0.069. The remaining three indices (color saturation, hue diversity, and spectral slope) were likewise examined for group differences; none differed significantly across conditions (all *p* > 0.05; detailed statistics in Supplementary Table S2). These results confirm that the three conditions were well-matched on the low-level visual features examined here, ruling out differences in luminance, contrast, visual complexity, color, and spatial-frequency content as alternative explanations for the condition effects reported below.

Beyond low-level visual matching, the three stimulus groups were matched on culturally relevant content attributes. In particular, all three groups contained comparable proportions of the three canonical ink-painting genres (landscape, figure, and bird-and-flower), and images bearing prominent text-like elements (e.g., inscriptions or seal marks) were excluded to prevent orthographic content from confounding cultural-attribute judgments. This content-level matching was intended to ensure that any observed condition differences reflect the generative origin of the stimuli rather than incidental variation in culturally salient features.

### 3.3 Visual display, luminance calibration, and stimulus synchronization

Stimuli were presented on a 24-inch liquid-crystal display (LCD; native resolution 1920 × 1,080 pixels; refresh rate 60 Hz; display model and product details are listed in the study’s reproducibility package) that was gamma-corrected via display calibration prior to data collection. Participants were seated at a chinrest-fixed viewing distance of 60 cm from the screen; under these conditions, each 1,024 × 1,024 pixel stimulus occupied approximately 29.6° × 29.6° of visual angle. Viewing was binocular under standard ambient illumination. Display model, resolution, refresh rate, viewing distance, and stimulus size / visual angle were recorded in line with the reporting recommendations of Lin et al. (2023a), Psychological Bulletin.

Because image pixel values do not necessarily map linearly onto physically displayed luminance, the grayscale linearity of the display was measured and the lookup table corrected using PsyCalibrator (Lin et al., 2023b), following the protocol described in the package documentation. The gamma-corrected display passed the standard contrast verification step recommended by PsyCalibrator prior to data collection in each session, and calibration reports were retained as part of the study’s reproducibility package.

The monitor’s pixel-response characteristics — that is, the time required for each liquid-crystal pixel to settle to its commanded luminance — were evaluated following the benchmark of Zhang et al. (2018). The display passed the suitability criterion described by Zhang et al. for sub-frame accurate visual stimulation; measured pixel-response rise and fall times were consistent with the 16.7-ms refresh window of the 60-Hz display, supporting reliable single-frame timing for stimulus onset.

Physical stimulus onset was synchronized with the EEG (BrainProducts actiCHamp, 512-Hz sampling) and 600-Hz eye-tracker (Tobii Pro Spectrum) by routing a transistor–transistor logic (TTL) trigger pulse from the stimulus-presentation computer at the moment a TrialHandler marker was issued. The TTL pulse was recorded simultaneously as a digital input on the EEG amplifier and as an asynchronous event marker on the eye-tracker’s data stream, yielding event-on alignment across the three modalities. The reliability of this synchronization scheme was verified prior to the experiment using a photoelectric sensor attached to the lower-right corner of the display; recorded onset latency jitter relative to the software trigger was below 1 ms across test trials (cf. Zhang et al., 2018).

### 3.4 Audience perception experiment

The perceptual experiment employed a multimodal measurement paradigm combining eye-tracking, semantic differential scaling, and EEG-based affective measurement to capture both implicit and explicit dimensions of audience response across the three stimulus conditions.

Eye-tracking data were collected using a Tobii Pro Spectrum remote infrared eye-tracker sampling at 600 Hz (the maximum available rate for this model). Prior to the experiment, a standard 9-point calibration procedure was administered for each participant, with a maximum accepted mean calibration error of 0.5° of visual angle; participants whose calibration did not meet this criterion were recalibrated before proceeding. Fixations were identified using a velocity-threshold dispersion algorithm (I-VT filter) with a velocity threshold of 30°/s and a minimum fixation duration of 60 ms. Trials in which the proportion of valid gaze samples fell below 75% of the total stimulus presentation period were excluded from analysis; no participant exceeded an exclusion rate of 15% of trials. Participants viewed each stimulus for a free-observation period of 8 s with no task instruction, allowing natural exploratory gaze behavior to unfold without cognitive interference. The primary index extracted was stationary gaze entropy (Shiferaw et al., 2019) — hereafter referred to as scan-path entropy — quantifying the spatial unpredictability of the viewing trajectory as:


$$H=-\sum_{i}p_{i}\log_{2}p_{i}$$

where p(i) denotes the probability of fixation occurrence within the i-th spatial region of a uniform grid partition of the image. For the purposes of this calculation, each 1,024 × 1,024 pixel stimulus image was partitioned into an 8 × 8 grid of 64 equal regions, yielding a maximum possible entropy of 6 bits. Higher entropy values indicate more dispersed and exploratory gaze patterns, which we interpret as a behavioral signature of expressive expansion engaging broader meaning-search processes.

The semantic differential instrument was developed specifically for this study through a three-stage procedure comprising item generation via expert interview, pilot testing with a convenience sample of 30 participants, and confirmatory factor analysis (CFA) to verify the four-factor structure. CFA was conducted using maximum likelihood estimation in R (lavaan package, version 0.6–12). The four-factor model demonstrated acceptable fit to the pilot data: χ^2^(164) = 218.43, *p* = 0.004; CFI (Comparative Fit Index) = 0.96; TLI (Tucker-Lewis Index) = 0.95; RMSEA (Root Mean Square Error of Approximation) = 0.058 (90% CI [0.031, 0.079]); SRMR (Standardized Root Mean Square Residual) = 0.061. All factor loadings were statistically significant (*p* < 0.001) and ranged from 0.61 to 0.84, indicating adequate convergent validity. Inter-factor correlations ranged from 0.21 to 0.47, supporting the discriminant validity of the four subscales. The final 20-item instrument was retained following removal of two items that exhibited cross-loadings exceeding 0.30 in the pilot CFA solution. The final scale comprised 20 bipolar adjective pairs distributed across four subscales: expressiveness, capturing the perceived dynamic and emotional energy of the work; authenticity, indexing the degree to which the work feels genuinely connected to the ink painting tradition; cultural resonance, measuring the strength of culturally learned associative activation; and innovativeness, reflecting perceived departure from conventional aesthetic norms. Each item was rated on a 0–100 continuous scale (presented as a visual-analog slider with integer steps), and subscale scores were computed as the mean of the constituent items following confirmation of acceptable internal consistency (α > 0.80 for all subscales).

EEG was recorded continuously throughout stimulus viewing using a BrainProducts actiCHamp 64-channel active electrode system with a sampling rate of 512 Hz, with electrodes arranged according to the standard 10–20 system. The online reference electrode was placed at CPz; data were subsequently re-referenced offline to the average of all 64 electrodes prior to further processing. Data were preprocessed offline using EEGLAB (v2024.0) running under MATLAB R2023b. Continuous data were bandpass filtered between 0.5 and 45 Hz (zero-phase Hamming-windowed FIR filter, order 1650), epoched from −500 ms to +8,000 ms relative to stimulus onset, and baseline-corrected using the 200 ms pre-stimulus interval. Artifact rejection was performed using joint probability criteria: epochs exceeding ±100 μV at any electrode were excluded (mean rejection rate: 4.7% ± 1.2% of trials across participants). Independent component analysis (ICA; runica algorithm, extended mode) was then applied to the remaining epochs. ICA components were visually inspected and those reflecting ocular artifacts (eye blinks and horizontal eye movements, identified by their characteristic frontal topography and correlation with EOG channels) and muscular artifacts (identified by their broadband high-frequency spectral profile). On average, 2.3 ± 0.8 components per participant were removed. Spectral power was estimated using Welch’s method (2-s Hanning window, 50% overlap, frequency resolution 0.5 Hz). Absolute power values were log-transformed (10 × log10) prior to statistical analysis. Beta suppression was computed as the difference in log-transformed beta power between the post-stimulus window (500–7,500 ms) and the pre-stimulus baseline (−200 to 0 ms). Baseline correction was applied using a 200 ms pre-stimulus window. Affective valence was indexed through the frontal alpha asymmetry score:


$$\mathrm{FAA}=\ln(F4_{\alpha })-\ln(F3_{\alpha })$$

where F3α and F4α denote alpha band (8–13 Hz) power at left and right frontal sites F3 and F4 respectively, with positive FAA values indicating approach-oriented positive affect. Cognitive engagement was additionally indexed through frontal beta power, computed as the mean beta band (13–30 Hz) power averaged across a bilateral frontal electrode cluster comprising F3, Fz, and F4:


$$P_{\beta }=\frac{1}{N}\sum_{k\in \beta }{|F(k)|}^{2}$$

where F denotes the Fourier coefficient at frequency bin k for electrode e, and N is the number of frequency bins in the beta band. For subsequent regression analyses, the beta suppression magnitude score was derived by reverse-coding the post-stimulus change in log beta power (multiplying by −1) so that higher values indicate stronger suppression; this coding direction is consistent with the interpretation in Tables 2, 3 and the Discussion. Higher beta suppression relative to the pre-stimulus baseline is interpreted, tentatively, as an indicator of deeper aesthetic immersion, consistent with prior neuroaesthetics literature, although the Limitations section notes that this inferential chain is not uniquely supported by the present data. It should be emphasized that oscillatory changes in specific frequency bands are not exclusive markers of aesthetic processing; beta suppression and frontal alpha asymmetry have been associated with a range of attentional, motor, and affective processes. Their interpretation here as indices of aesthetic immersion and approach-oriented affect follows convergent prior evidence rather than a one-to-one neural mapping.

**Table 2:** EEG affective neural responses by stimulus condition (all participants).

Measure	Traditional	AI-Generated	Digital Non-AI	F (2,238)	η^2^p [95% CI]
FAA score	0.151 ± 0.024	0.254 ± 0.026***	0.175 ± 0.024***	1851.12	0.940 [0.927, 0.949]
Beta suppression magnitude (dB)	0.903 ± 0.113	1.550 ± 0.109***	1.033 ± 0.110***	4390.85	0.974 [0.968, 0.978]

Asterisks indicate significant pairwise differences relative to the traditional condition (Tukey HSD corrected), ***p < 0.001. Bonferroni correction was applied across the two simultaneous EEG ANOVAs, yielding an adjusted threshold of α = 0.025; both omnibus effects remained significant under this criterion.

**Table 3:** Multiple regression models predicting expressiveness and cultural authenticity ratings from implicit measures, by expertise group (AI-generated condition only).

Predictor	Expressiveness professional β	Expressiveness non-professional β	Cultural authenticity professional β	Cultural authenticity non-professional β
Scan-path entropy	0.154	0.477	0.061	0.261
FAA score	0.385	−0.569	−0.155	0.619
Beta suppression magnitude	−0.153	0.227	−0.757	0.638
Model R^2^ (adj.)	−0.03	0.04	−0.00	−0.01

Beta values are standardized regression coefficients. Standardized regression coefficients (β); none of the coefficients reached statistical significance (all *p* > 0.10). All predictors z-scored within group prior to analysis. Regressions were computed at the participant level (*N* = 60 per group) using each participant’s mean across the 20 AI-generated artworks. Beta suppression magnitude is the reverse-coded beta power change (higher = stronger suppression). Predictor × Expertise interaction terms, estimated in the combined sample (*N* = 120), are reported in Supplementary Table S3.

The experiment was administered in a single session of approximately 90 min. Stimulus presentation order was fully counterbalanced across participants using a Latin square design, and a 2-s blank inter-stimulus interval was inserted between trials to allow neural and oculomotor baselines to recover. Participants were instructed to view each image naturally and to complete the semantic differential ratings immediately following each stimulus before proceeding to the next trial.

### 3.5 Participants

A total of 120 participants were recruited and allocated equally across two expertise groups: 60 art professionals and 60 non-professionals. Art professionals were defined as individuals holding at least a bachelor’s degree in fine arts, visual arts, or a closely related discipline, with documented formal training or active professional practice specifically in Chinese ink painting — encompassing brush technique, compositional philosophy, and culturally grounded aesthetic criteria. Expertise in adjacent domains such as art history, digital illustration, or other painting traditions without specific ink painting training did not qualify for inclusion in this group. At recruitment, all professional candidates were required to submit verifiable documentation of their ink painting training, in the form of either official transcripts specifying ink painting coursework, a letter of verification from a supervising instructor or institution, or a portfolio of original ink painting works evaluated for technical and stylistic adequacy by two members of the research team who were themselves trained ink painting practitioners. Candidates whose submitted materials did not demonstrate sufficient engagement with the core technical and aesthetic conventions of the Chinese ink painting tradition were excluded. Within the professional group, participants reported a mean of 7.98 ± 2.69 years of formal training specifically in Chinese ink painting (range: 4–15.3 years), with a minimum of 4 years required for inclusion. Non-professionals reported no formal arts training of any kind and a mean of 0.50 ± 0.43 years of self-directed engagement with visual art production. Non-professionals were defined as individuals with no formal arts education and no self-reported regular engagement with visual art production, recruited through university general subject pools and community postings.

The two groups were matched on age range (18–35 years), gender distribution (balanced at 50% female within each group), and normal or corrected-to-normal vision. All participants were right-handed, as verified by self-report at screening. All participants reported no history of neurological or psychiatric disorder, no color vision deficiency, and no prior participation in studies involving ink painting stimuli. To control for potential attitude confounds on authenticity judgments, participants were additionally screened for prior exposure to AI-generated art: individuals who reported professional or sustained recreational engagement with AI image generation tools (e.g., regular use of Stable Diffusion, Midjourney, or equivalent platforms exceeding five hours per week) were excluded. Casual or incidental prior exposure was permitted and recorded for use as a covariate in sensitivity analyses.

Within each expertise group, participants were further randomly assigned to one of three stimulus presentation orders generated by the Latin square counterbalancing scheme described in Section 3.4, yielding 20 participants per order cell and ensuring that order effects were fully orthogonal to the expertise factor. Sample size was determined *a priori* through power analysis targeting 80% power to detect a medium effect size (*f* = 0.25) at α = 0.05 for the primary expertise-by-condition interaction in a mixed-design ANOVA, which indicated a minimum requirement of 108 participants and was rounded up to 120 to accommodate potential attrition.

All participants provided written informed consent prior to enrollment, with explicit notification that participation was voluntary and that consent could be withdrawn at any time without penalty and without requirement to provide a reason. Withdrawal prior to data analysis would result in complete removal of the participant’s data from the dataset. All procedures involving human participants—including eye-tracking, EEG recording, and self-report measures—were conducted in accordance with the Declaration of Helsinki, and the study protocol was reviewed and approved by the Ethics Review Board of the School of Physical Education, Shandong Normal University (IRB No. SDNUTYDW2024053). Data collection was conducted at Shandong Normal University.

### 3.6 Statistical analysis

All statistical analyses were conducted in R version 4.4.0 (R Core Team, 2024) using the ez (Lawrence, 2016), emmeans (Lenth, 2023), lme4 (Bates et al., 2015), lmerTest (Kuznetsova et al., 2017), and effectsize (Ben-Shachar et al., 2020) packages. For the condition-level comparisons in Sections 4.1, 4.3, and 4.4, one-way and repeated-measures ANOVAs were conducted as described above, with Bonferroni correction applied to control the Type I error rate across simultaneous tests. Crossed mixed-effects models incorporating random intercepts for both participants and artworks constituted the primary analytical framework for the principal outcomes — expressiveness, cultural authenticity, scan-path entropy, and the EEG indices — because responses were nested within both participants and artworks (120 participants, 20 artworks per condition): Outcome ∼ Condition × Expertise + (1|Participant) + (1|Artwork). Random slopes for Condition at the participant level were added where model convergence and the structure of the data permitted, and the two models were compared by likelihood-ratio tests. The mixed-effects results, which generalize beyond the particular stimuli selected, are reported in Section 4.6 and Table 4. For comparability with prior literature and ease of interpretation, conventional repeated-measures ANOVA statistics are additionally reported in Sections 4.1–4.4 and were corroborated by the primary mixed-effects results. Complete R scripts and a reproducible analysis pipeline are deposited alongside this manuscript.

**Table 4:** Crossed mixed-effects model estimates for the principal outcomes (fixed effects; random intercepts for participants and artworks).

Outcome	Fixed effect	Estimate (b)	SE	df	t	*p*	ICC artwork
Expressiveness	(Intercept)	55.642	0.562	135.5	99.05	<0.001	0.043
Expressiveness	Condition [AI vs. Trad]	9.815	0.655	74.8	14.99	<0.001	—
Expressiveness	Expertise [Prof]	−0.883	0.558	200.4	−1.58	0.115	—
Expressiveness	Condition × Expertise	0.383	0.467	7019.0	0.82	0.412	—
Cultural authenticity	(Intercept)	77.409	0.741	138.6	104.42	<0.001	0.048
Cultural authenticity	Condition [AI vs. Trad]	−38.859	0.848	72.7	−45.82	<0.001	—
Cultural authenticity	Expertise [Prof]	−0.224	0.738	184.9	−0.30	0.762	—
Cultural authenticity	Condition × Expertise	11.820	0.574	7019.0	20.59	<0.001	—
Scan-path entropy	(Intercept)	3.304	0.137	147.7	24.17	<0.001	0.019
Scan-path entropy	Condition [AI vs. Trad]	0.656	0.071	77.6	9.26	<0.001	—
Scan-path entropy	Condition × Expertise	0.168	0.054	7019.0	3.14	0.002	—
FAA score	(Intercept)	0.151	0.005	126.3	31.49	<0.001	0.061
FAA score	Condition [AI vs. Trad]	0.103	0.006	69.4	18.32	<0.001	—
Beta suppression magnitude	(Intercept)	0.920	0.022	121.3	41.42	<0.001	0.075
Beta suppression magnitude	Condition [AI vs. Trad]	0.641	0.026	66.8	24.45	<0.001	—

Models were fitted by REML with lme4; *p* values from Satterthwaite’s approximation (lmerTest). ICC_artwork is the intraclass correlation attributable to the artwork-level random intercept. Random slopes for Condition at the participant level were added where convergence permitted; the reduced and full models were compared by likelihood-ratio tests (complete outputs in the Supplementary Materials).

## 4 Results

### 4.1 Quantitative evidence of expressive expansion

Comparison of the three stimulus conditions on the expressive expansion dimensions revealed a consistent pattern in which AI-generated ink works scored significantly higher than traditional originals on expressiveness and innovativeness, while traditional originals led on cultural resonance. One-way repeated-measures ANOVAs conducted for the three dimensions measured in all conditions confirmed that condition effects were statistically significant: expressiveness, *F* (2, 238) = 966.86, *p* < 0.001; innovativeness, *F* (2, 238) = 3436.57, *p* < 0.001; cultural resonance, *F* (2, 238) = 1883.74, *p* < 0.001. To control for Type I error inflation across the three simultaneous ANOVAs, a Bonferroni correction was applied, yielding an adjusted significance threshold of α = 0.0167; all three omnibus effects remained significant under this criterion. Post-hoc Tukey Honestly Significant Difference (HSD) tests identified the specific pairwise contrasts driving these omnibus effects, with effect sizes and 95% confidence intervals reported in Table 5. These condition differences pertain to the specific stimulus sets and experimental conditions examined here and should not be generalized to AI-generated ink painting as a class; they were corroborated by the primary crossed mixed-effects analyses reported in Section 4.6 (Table 4).

**Table 5:** Mean scores (±SD) across the three expressive expansion dimensions (0–100 scale) by stimulus condition.

Dimension	Traditional (M ± SD)	AI-generated (M ± SD)	Digital non-AI (M ± SD)	AI vs Trad (d [95% CI])	AI vs Dig (d [95% CI])	η^2^p [95% CI]
Expressiveness	55.20 ± 3.12	65.21 ± 2.90***	59.54 ± 3.16***	3.86 [3.34, 4.38]	2.33 [1.98, 2.67]	0.890 [0.868, 0.908]
Innovativeness	48.88 ± 3.60	71.16 ± 3.39***	57.78 ± 3.49***	7.31 [6.35, 8.24]	4.49 [3.88, 5.08]	0.967 [0.960, 0.972]
Cultural Resonance	74.32 ± 3.41	48.37 ± 6.74***	60.19 ± 4.46***	−4.36 [−4.93, −3.77]	−2.91 [−3.32, −2.50]	0.941 [0.928, 0.950]

Effect sizes are Cohen’s *d* for paired comparisons (positive values indicate higher scores for the AI-generated condition); *η*_p_^2^ is partial eta-squared for the omnibus *F* with 95% confidence intervals estimated via the non-central *F* distribution. Asterisks indicate significant pairwise differences relative to the traditional condition (Tukey HSD corrected), ***p < 0.001.

The full pattern of condition means and confidence intervals across all three dimensions is presented in Figure 2.

**Figure 2:**
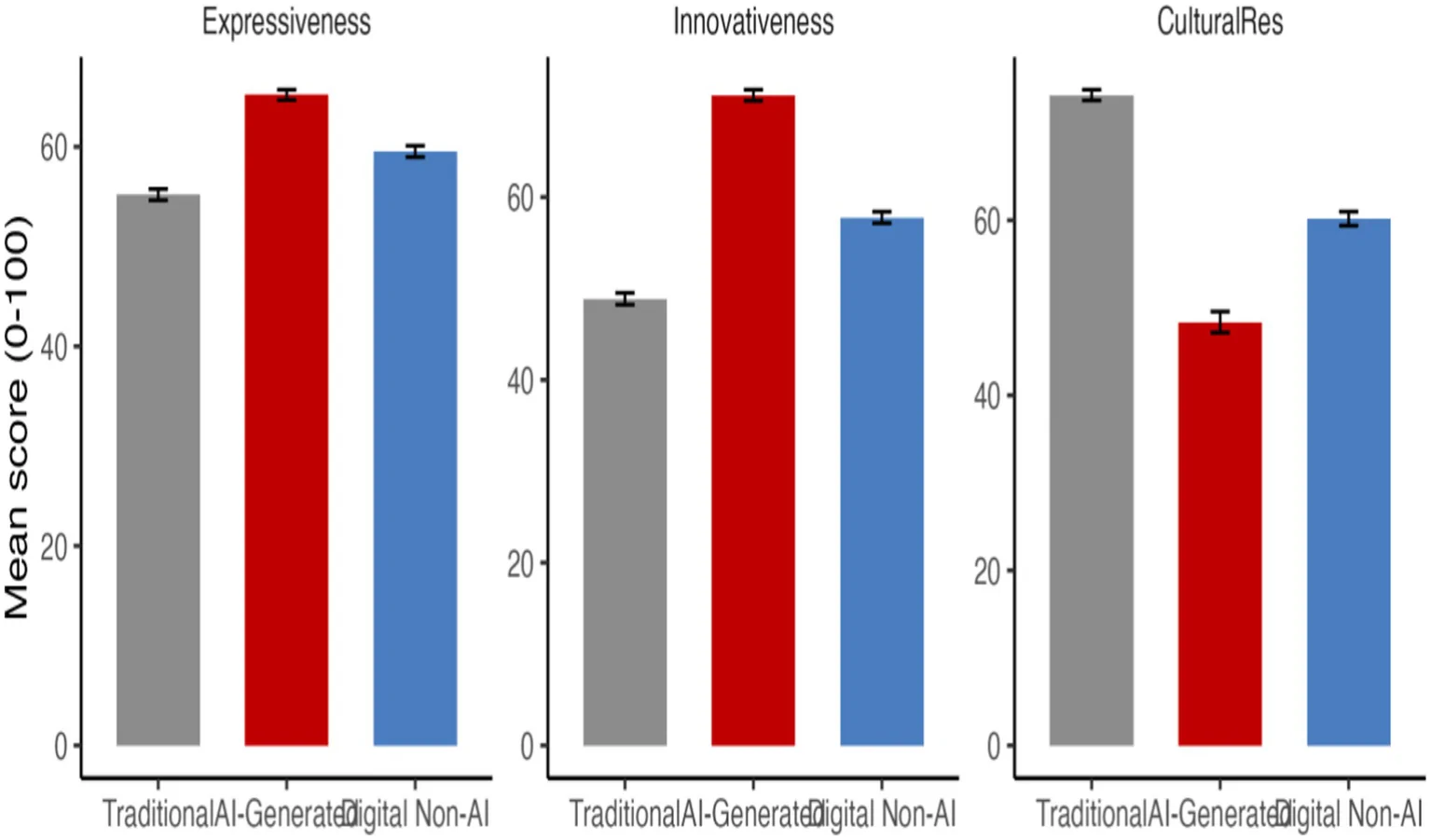
Expressive expansion dimension scores by stimulus condition. Mean scores across the three expressive expansion dimensions (expressiveness, innovativeness, cultural resonance; 0–100 scale) for three stimulus conditions (traditional originals, AI-generated, digital non-AI), with 95% confidence intervals. Asterisks indicate significant pairwise differences relative to the traditional condition: ***p < 0.001.

### 4.2 Audience perception differentiation

Analysis of semantic differential ratings revealed a systematic divergence between professional and non-professional audiences that was dimension-specific rather than global, indicating that expertise shapes aesthetic response in selective and theoretically meaningful ways. A two-way mixed ANOVA with expertise (professional vs. non-professional) as a between-subjects factor and stimulus condition as a within-subjects factor was conducted separately for each subscale, with results summarized in Figure 3. The two-way ANOVA results were corroborated by the primary crossed mixed-effects analyses reported in Section 4.6 (Table 4).

**Figure 3:**
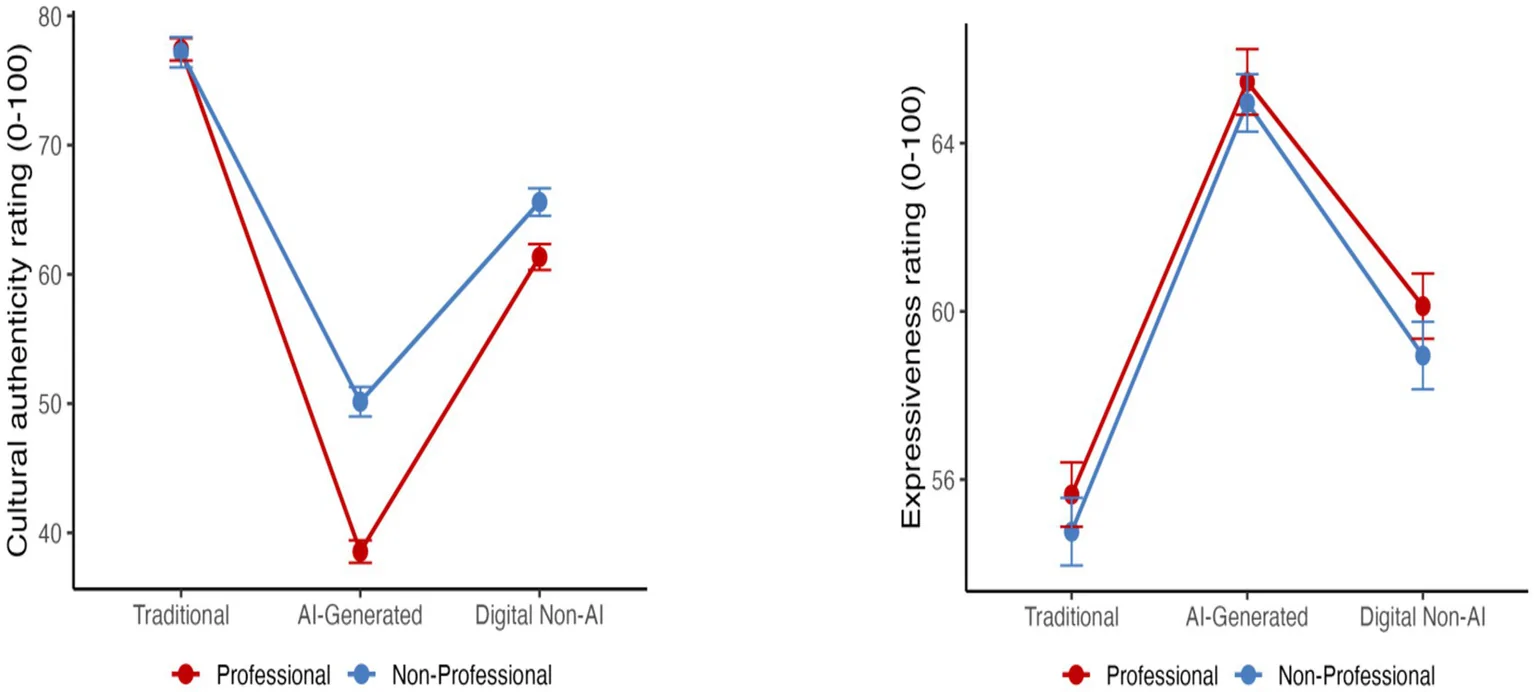
Semantic differential ratings for cultural authenticity and expressiveness across expertise groups and stimulus conditions. Mean semantic differential ratings (0–100 scale) for cultural authenticity and expressiveness subscales across expertise groups (professional vs. non-professional) and stimulus conditions (traditional originals, AI-generated, digital non-AI), with 95% confidence intervals, ***p < 0.001.

Semantic differential ratings revealed a two-cluster pattern: expertise differentiated responses on culturally grounded dimensions (authenticity and cultural resonance) but not on perceptually driven dimensions (expressiveness and innovativeness). Two-way mixed ANOVAs with expertise as the between-subjects factor and stimulus condition as the within-subjects factor revealed a significant main effect of Condition for all four subscales, a significant main effect of Expertise for cultural authenticity and cultural resonance but not for expressiveness or innovativeness, and — critically for the dual-pathway account — a significant Condition × Expertise interaction for cultural authenticity and cultural resonance but not for expressiveness or innovativeness (F statistics in Table 6). Follow-up simple-effects tests of the Expertise contrast within each condition, corrected with the Tukey–Kramer procedure, showed that the professional–non-professional difference on the cultural dimensions was significant only within the AI-generated condition; detailed statistics are presented in Table 6.

**Table 6:** Two-way mixed ANOVA of semantic differential ratings with expertise (professional vs. non-professional) as the between-subjects factor and stimulus condition (traditional, AI-generated, digital non-AI) as the within-subjects factor.

Subscale	Professional (M ± SD)	Non-professional (M ± SD)	Expertise *F* (1,118)	Condition *F* (2,236)	Expertise × Condition F(2,236)	*p*	η^2^p [95% CI]
Cultural Authenticity	38.55 ± 3.44	50.15 ± 4.51	62.37	6426.95	209.02	<0.001	0.346 [0.215, 0.463]
Expressiveness	65.46 ± 3.08	64.96 ± 2.70	3.04	967.71	1.11	0.084	0.025 [0.000, 0.105]
Cultural Resonance	42.61 ± 3.58	54.12 ± 3.39	118.57	4977.95	196.47	<0.001	0.501 [0.378, 0.599]
Innovativeness	70.76 ± 3.41	71.56 ± 3.34	0.47	3435.72	0.97	0.495	0.004 [0.000, 0.056]

Group means (±SD) are reported for the AI-generated condition; the same pattern holds qualitatively across the other conditions (see Figure 3). Simple effects of Expertise within each Condition were tested following significant Condition × Expertise interactions, with Tukey–Kramer correction. Exact F statistics, degrees of freedom, and exact *p* values are reported in Table 6 and in the R analysis outputs provided in the Supplementary Materials. η^2^p is partial eta-squared with 95% confidence intervals estimated via the non-central F distribution. To control for Type I error inflation across four simultaneous expertise-effect tests, a Bonferroni-corrected threshold of α = 0.0125 was applied. η^2^p values are reported with 95% confidence intervals estimated via the non-central F distribution.

### 4.3 Eye movement pattern differences

Analysis of eye-tracking data revealed that stimulus condition exerted a significant effect on scan-path entropy, with AI-generated ink works consistently eliciting more spatially dispersed gaze behavior than either traditional originals or digital non-AI works. A one-way repeated-measures ANOVA confirmed a significant main effect of condition on scan-path entropy, *F* (2, 238) = 18.43, *p* < 0.001, η^2^p = 0.134 [95% CI: 0.061, 0.213]. Pairwise comparisons were conducted using Tukey HSD correction. The AI vs. traditional contrast yielded d = 0.39 [95% CI: 0.20, 0.57]; the AI vs. digital non-AI contrast yielded d = 0.49 [95% CI: 0.30, 0.68]. AI-generated works elicited significantly higher scan-path entropy than both comparison conditions, consistent with the hypothesis that expressive expansion activates broader meaning-search behavior. Within the professional group, inter-individual variability in entropy was notably higher for AI-generated works. Key eye-tracking statistics are presented in Table 7. Because responses were nested within both participants and artworks, the condition effect on scan-path entropy was corroborated by the primary crossed mixed-effects analysis (Section 4.6, Table 4).

**Table 7:** Scan-path entropy by stimulus condition (all participants).

Measure	Traditional	AI-generated	Digital non-AI	*F* (2,238)	η^2^p [95% CI]
Scan-path entropy (bits)	3.27 ± 0.33	4.01 ± 1.91***	3.54 ± 1.00	18.43	0.134 [0.061, 0.213]

For the primary measure, any trial-level data excluded by the quality criteria described in Section 3.4 were accommodated by the crossed mixed-effects models reported in Section 4.6. Asterisks indicate significant pairwise differences relative to the traditional condition (Tukey HSD corrected), ***p < 0.001.

The full distribution of scan-path entropy values across conditions and expertise groups is presented in Figure 4.

**Figure 4:**
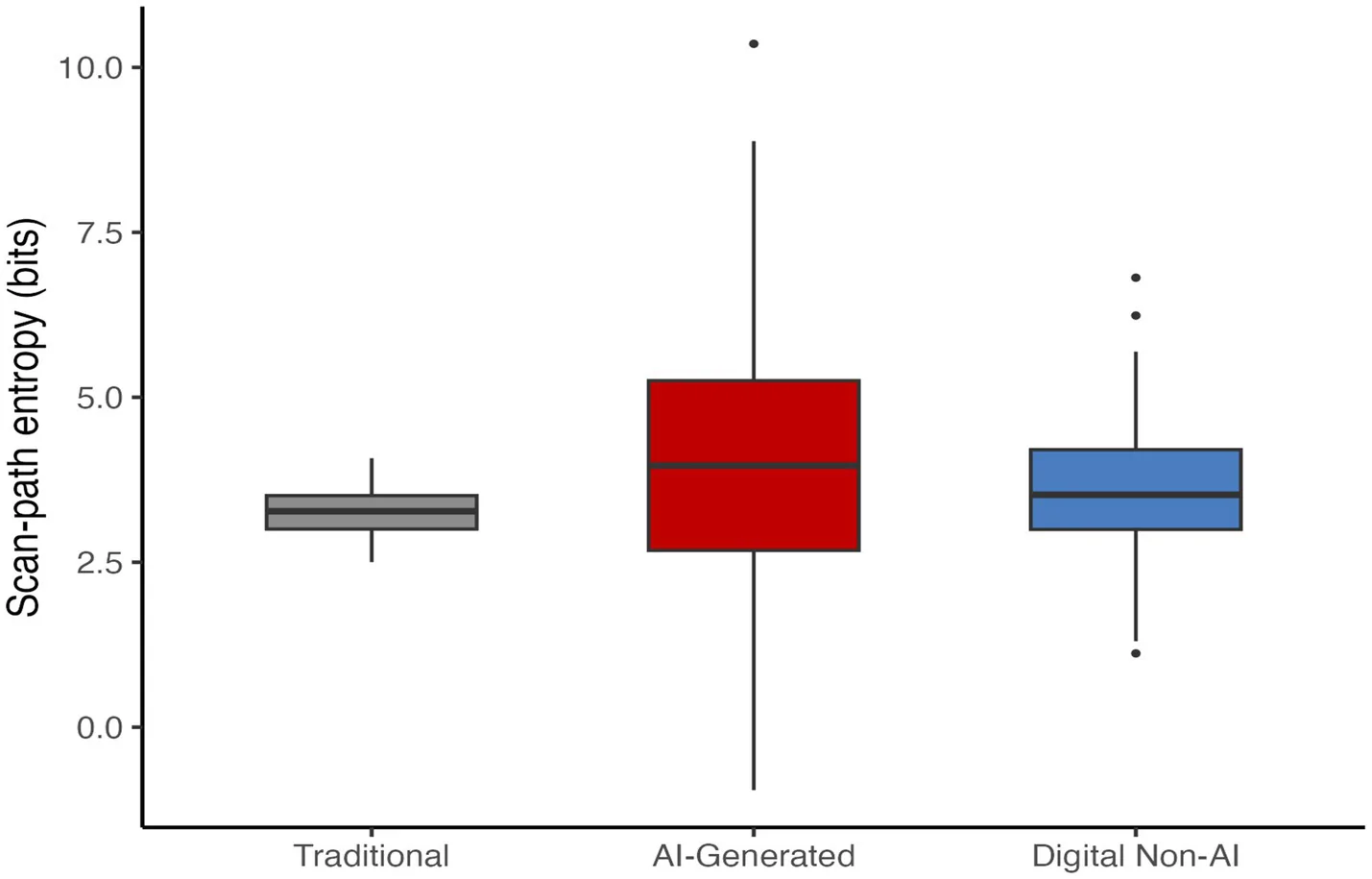
Scan-path entropy distributions across stimulus conditions and expertise groups. Box plots show median, interquartile range, and individual data points. Box plots are used here to highlight the distributional properties (including skew and outliers) that are obscured by mean-based displays.

### 4.4 Affective neural responses

EEG analysis revealed that stimulus condition significantly modulated both frontal alpha asymmetry and beta band power. AI-generated works elicited the strongest beta suppression and the highest frontal alpha asymmetry scores relative to both comparison conditions, a pattern suggestive of — but not uniquely attributable to — deeper aesthetic immersion and approach-oriented affect (see Limitations). Full neural response statistics are presented in Table 2. These EEG effects were corroborated by the primary crossed mixed-effects analyses reported in Section 4.6 (Table 4).

The full pattern of FAA and beta suppression values across conditions is presented in Figure 5.

**Figure 5:**
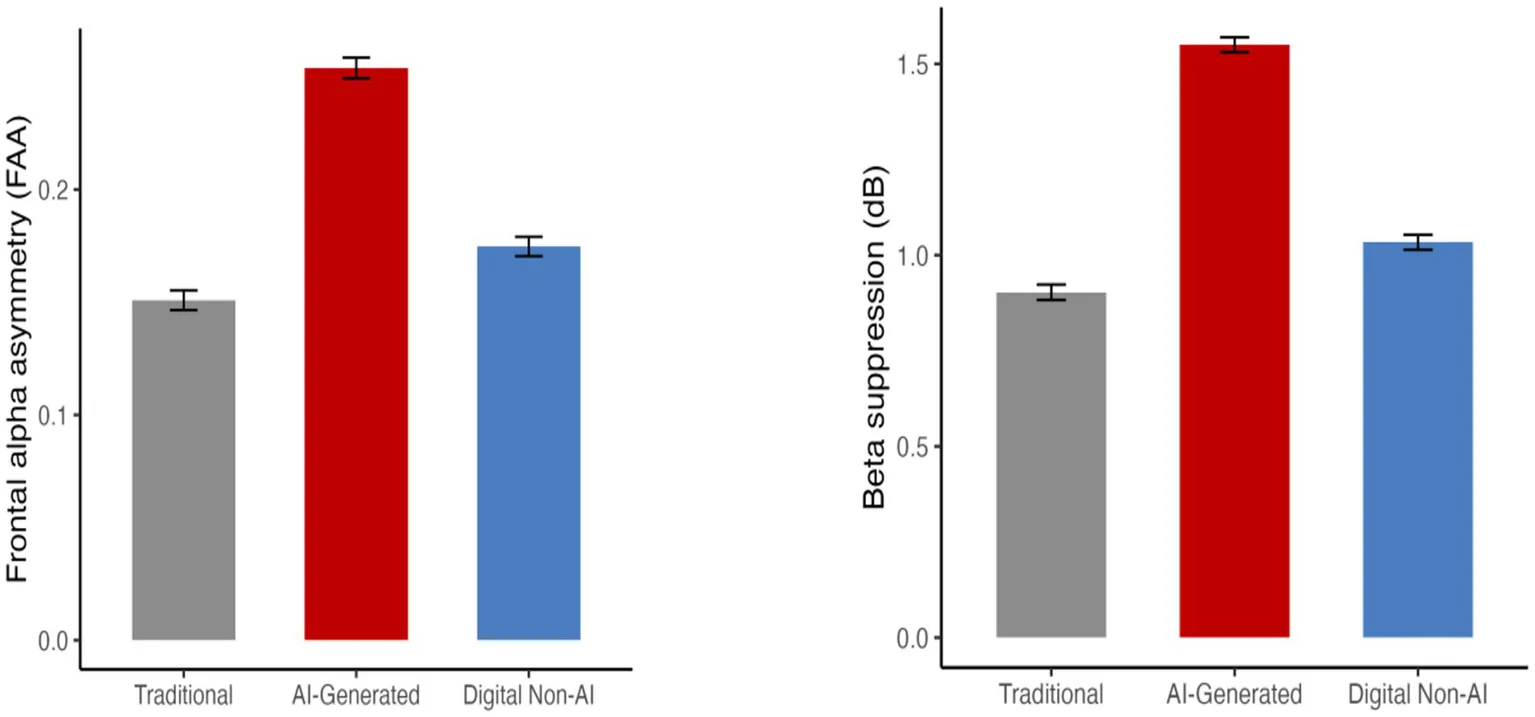
EEG affective neural responses across stimulus conditions. Error bars = 95% CI; ***p < 0.001.

### 4.5 Cross-modal associative analyses

To examine whether implicit behavioral and neural indices of aesthetic engagement were systematically linked to explicit aesthetic ratings — as predicted by H5 — and to characterize the moderating role of expertise across measurement modalities, multiple linear regression analyses were conducted on the AI-generated stimulus condition. To avoid violating the independence assumption that would follow from analyzing trial-level observations (each participant rated each of the 20 AI-generated artworks), all regressions were conducted at the participant level, using each participant’s mean across the 20 AI-generated artworks (*N* = 60 per expertise group). In addition, to directly test whether the predictive relationships differed between expertise groups, the models were re-estimated in the combined sample (*N* = 120) with predictor × Expertise interaction terms.

In each participant-level regression model, scan-path entropy, FAA score, and beta suppression magnitude served as predictor variables; beta suppression magnitude was reverse-coded from the post-stimulus beta power change (multiplied by −1) so that higher values indicate stronger suppression, making the coding direction consistent with the operational definition in Section 3.4 and with the interpretation in Tables 2, 3 and the Discussion. Expressiveness rating and cultural authenticity rating were entered as dependent variables in separate models. All predictors were standardized prior to analysis (z-scored within group) to permit direct comparison of standardized regression coefficients (β) across predictors. Model fit was evaluated using adjusted R^2^, and predictor significance was assessed at α = 0.05. Predictor × Expertise interaction terms were tested in the combined-sample models (*N* = 120). The interaction estimates, standard errors, 95% confidence intervals, t statistics, and *p* values are reported in Supplementary Table S3 and summarized below. Statistical analyses were conducted in R 4.4.0 (R Core Team, 2024) using the lme4 (Bates et al., 2015) and effectsize (Ben-Shachar et al., 2020) packages.

The regression results did not support H5: across both expertise groups, none of the implicit measures — scan-path entropy, frontal alpha asymmetry, or beta suppression magnitude — significantly predicted expressiveness ratings (all *p* > 0.10), and the three-predictor models accounted for negligible variance in expressiveness (adjusted R^2^ = −0.03 to 0.04). Likewise, none of the implicit measures significantly predicted cultural authenticity ratings in either group (all *p* > 0.10; adjusted R^2^ ≈ 0). Formal tests of predictor × Expertise interactions in the combined sample (*N* = 120) likewise revealed no significant differences between expertise groups for any predictor–outcome combination (all *p* > 0.05; full estimates, standard errors, 95% confidence intervals, and *p* values in Supplementary Table S3). The absence of systematic associations between the implicit behavioral and neural indices and the explicit ratings in the present data means that H5 — and any interpretation linking the two processing routes through within-individual correlations — is not supported; the dual-pathway account therefore rests on the dissociating pattern of condition and expertise effects reported in Sections 4.1–4.4 rather than on cross-measure associations. Because the regressions were conducted at the participant level (*N* = 60 per group; each participant’s mean across the 20 AI-generated artworks), the restricted between-participant variance in the standardized ratings may have limited the power of these tests.

### 4.6 Crossed mixed-effects analyses (primary analytical framework)

Because each participant rated each stimulus and the 20 artworks within each condition constituted a sample from a larger class of stimuli, the primary analytical framework of the study was a series of crossed mixed-effects models that included random intercepts for both participants and artworks: Outcome ∼ Condition × Expertise + (1|Participant) + (1|Artwork), where the outcome was, in turn, expressiveness, cultural authenticity, scan-path entropy, frontal alpha asymmetry, and beta suppression magnitude. Models were fitted by restricted maximum likelihood (lme4; Bates et al., 2015); *p* values were obtained via Satterthwaite’s approximation (lmerTest; Kuznetsova et al., 2017). Random slopes for Condition at the participant level were added where convergence was achieved, and the two models were compared by likelihood-ratio tests. Effect sizes (Cohen’s d) for significant contrasts were computed from the estimated marginal means (emmeans). Full model outputs are reported in Table 4.

The crossed mixed-effects analyses — the primary analytical framework of the study — confirmed the principal findings reported in Sections 4.1–4.4 after simultaneously accounting for participant- and artwork-level variability. AI-generated works received significantly higher expressiveness ratings (b = 9.815, SE = 0.655, t = 14.99, *p* < 0.001) and higher scan-path entropy (b = 0.656, SE = 0.071, t = 9.26, *p* < 0.001) than traditional originals, with digital non-AI works falling between; professional viewers rated AI-generated works as significantly less culturally authentic than non-professionals (Expertise × Condition interaction: b = 11.820, SE = 0.574, t = 20.59, *p* < 0.001). Comparable patterns were observed for the EEG indices (Table 4). The artwork-level random intercept accounted for a non-negligible proportion of the variance in all models (ICC_artwork = 0.019–0.075), indicating that responses varied systematically across artworks. This nonzero artwork-level ICC does not by itself establish that the condition effects generalize beyond the selected stimuli; rather, generalizability is supported to the extent that the fixed effects and interactions of interest remain statistically supported once artwork-level variability is included in the model, as reported above and in Table 4. Because these models generalize over both participants and artworks, they address the concern that the ANOVA-based results might be driven by a subset of the selected images. Full model outputs — fixed-effect estimates, degrees of freedom, ICC values, random-effect specifications, and likelihood-ratio tests of model comparison — are provided in Table 4 and in the R analysis scripts (see Supplementary materials).

## 5 Discussion

The present findings are consistent with the interpretation that AI-created ink art may generate compositional uncertainties, tonal transitions, and spatial conflicts that show certain parallels with the ink painting philosophy of ‘contingency/accident’ (偶然性). Whether this constitutes a genuine aesthetic equivalent to human-mediated chance remains an open question that the present data cannot resolve. Ink painting theory has long proposed that compelling aesthetics can result from the deliberate relinquishment of control to the medium, whereby ink seeps, puddles, and spreads beyond the original intentions of the artist. The latent diffusion model bears a structural analogy to this process: random noise is successively transformed into meaningful shapes through denoising, and the resulting images may occupy regions of the artistic space that are difficult to reach through controlled human brushwork (Garcia, 2025). There is a discernible pattern underlying these results: under the present experimental conditions, AI-generated works received significantly higher viewer ratings on expressiveness and innovativeness than their traditional counterparts, even though the former lacked aesthetic intention in any conventional sense. This finding is specific to the perceptual-affective route of audience response under the present experimental conditions and should not be generalized to broader claims about artistic merit or creative superiority.

This result bears several significant theoretical implications regarding the relationship between generative AI and human creativity. Based on recent findings from large scale experiments, one can assume that text to image AI augments human creativity in terms of productivity while causing paradoxical contraction in terms of diversity of creativity among artists as they adopt similar approaches to their creations (Zhou and Lee, 2024). The present results extend this observation by providing preliminary evidence that the stochasticity involved in the generation process may produce viewer-perceived expressive effects — specifically on the dimensions of expressiveness and innovativeness — that differ systematically from those elicited by works created within the constraints of the traditional medium. It should be emphasized that this finding pertains exclusively to audience perception under the present experimental conditions and does not constitute a claim about the generative capacity of AI systems relative to trained artists (Doshi and Hauser, 2024). It should be acknowledged, however, that this interpretation is not without contestation. A competing view holds that the apparent expressive freedom of latent diffusion outputs is not a genuine aesthetic affordance but rather a form of statistical averaging across the training distribution — what some critics have described as the recombination of existing human creativity rather than its transcendence (Doshi and Hauser, 2024). Under this interpretation, what appears as constraint liberation may more accurately reflect the model’s failure to internalize the intentional logic that governs human brushwork, producing outputs that superficially resemble aesthetic risk-taking without the underlying deliberative process that gives such risk meaning within the ink painting tradition. The present data cannot definitively adjudicate between these two interpretations, and future work combining process-tracing methods with reception studies will be needed to resolve this ambiguity.

Notwithstanding these observations, the audience perception data suggest a two-sided pattern of responses. On the one hand, both professional and non-professional viewers rated the AI-generated works as highly expressive. On the other hand, professional viewers rated these same works as substantially less culturally authentic than did non-professionals (Li, 2025). This divergence is consistent with the theoretical view that aesthetic appreciation can proceed largely independently of cultural knowledge, whereas cultural legitimacy judgments require familiarity with tradition-specific criteria (Mutter and Hübner, 2024). Consistent with prior research, the present data suggest that expertise may increase sensitivity to both the intentionality of the work and its culture-specific aspects, potentially widening the gap between perceptual engagement and explicit cultural validation (Messingschlager and Appel, 2025). What emerges is a characteristic difference between expressiveness ratings and perceived cultural-authenticity ratings — a pattern we interpret, in line with the dual-pathway framework, as a dissociation between the perceptual-affective and cultural-evaluative routes. We emphasize that cultural anxiety or evaluative concern was not directly measured in the present study, and the characterization of the two routes remains inferential.

This pattern can be productively approached using the concept of dissociation. Aura, a term proposed initially by Benjamin in his analysis, and later re-examined in light of AI by modern research, states that the value of an artwork depends on its conditions of existence and production, including both the artist’s presence and uniqueness of the creative process (Park, 2025). In other words, AI-generated ink paintings may challenge traditional ontology in fundamental ways, since they may be perceived as comparable or superior to traditional paintings on certain aesthetic dimensions while being regarded as inauthentic with respect to the art tradition they reference (Salas Espasa and Camacho, 2025). Semi-aura is the term better describing this unique situation, as opposed to total acceptance or complete rejection of the creative potential of artificial intelligence (Malecki et al., 2025). The present findings indicate that sensitivity to the ontological ambiguity inherent in AI-produced artwork was significantly more pronounced among professional viewers than non-professionals under the current experimental conditions. Whether this pattern reflects a stable cross-situational disposition or is sensitive to contextual and attributional factors remains a question for future research.

A methodological consideration that bears directly on the interpretation of these findings concerns the blind viewing paradigm employed in the present study. Participants were not informed of the authorship category — traditional, AI-generated, or digital non-AI — of any stimulus throughout the experimental session. Under this design, the responses recorded are likely to reflect primarily the perceptual and affective properties of the stimuli themselves, although the possibility that some participants inferred authorship from visual or stylistic cues cannot be excluded (see below). This design choice is consistent with the implicit measurement logic of the dual-pathway framework, which treats eye-tracking and EEG indices as capturing perceptual-affective processing that precedes and operates independently of explicit authorship knowledge. An important limitation of the blind viewing design is that it did not assess whether participants could infer AI authorship from visual or stylistic cues alone. Professional ink painters may have detected AI-like artifacts, implausible brushwork, or compositional irregularities, meaning their lower authenticity ratings could reflect source detection rather than, or in addition to, tradition-based authenticity criteria. Because source attribution was not measured, the present data cannot distinguish between these two pathways. Furthermore, the blind viewing design imposes an important boundary condition on generalizability: the results cannot be directly extrapolated to real-world contexts in which AI authorship is disclosed, as attribution knowledge has been shown to substantially modulate ratings (Di Dio et al., 2023; Messingschlager and Appel, 2025). The lower cultural authenticity ratings observed among professional viewers in the present study might be expected to persist or even to amplify under disclosed-authorship conditions, given prior evidence that disclosure reduces authenticity ratings (Di Dio et al., 2023; Messingschlager and Appel, 2025); however, disclosure could also alter judgments in more complex ways, and the present blind-viewing data do not permit us to determine which scenario obtains. Future research should directly compare viewer responses under blind and disclosed authorship conditions to disentangle perceptual effects from attribution effects, and to examine whether this dissociation is amplified, attenuated, or qualitatively altered when AI authorship is made explicit.

Additional nuance emerges from the neural findings. Given the association between beta band suppression and aesthetic immersion reported in prior work, one possible interpretation is that AI-generated artworks induced stronger aesthetic immersion than traditional works under the present experimental conditions (Welter and Lotte, 2024). The neural findings are also consistent with the interpretation that participants showed more positive approach-related affect while viewing AI-generated ink paintings, although this pattern was not consistent across all conditions (Shemyakina et al., 2024). Overall, these results are consistent with the interpretation that the perceptual-affective properties of algorithmically generated ink paintings engage cognitive processing that is, at least in part, independent of cultural evaluative criteria — though the causal direction of this relationship cannot be established from the present cross-sectional data. According to dual-process models, affective responses and explicit evaluations of artwork can be independent under ambiguous ontological circumstances (Eom and Kim, 2025). Within the embodied cognition framework, it is possible that the lack of a starting gesture in algorithmic ink painting would impede the typical motor resonance component of the experience, leading to different individual efforts to identify embodied markers within algorithmic ink paintings (Eom and Kim, 2025).

The current study, through its engagement with existing literature on the use of artificial intelligence in understanding artworks, supports as well as advances the emerging pattern. In line with previous research, the present results are consistent with the well-documented pattern of lower cultural authenticity ratings for AI-generated works. Importantly, however, the finding that expressiveness ratings for AI-generated works were comparably elevated across both expertise groups — under blind viewing conditions in which authorship was withheld — represents a meaningful qualification of perspectives that characterize AI art reception as uniformly negative, at least with respect to perceptually driven aesthetic dimensions (Messingschlager and Appel, 2025). The field of Chinese ink painting is particularly relevant for advancing theories surrounding the use of AI due to the emphasis placed on chance elements, thereby providing a cultural context in which stochastic processes involved in AI can be viewed positively—unlike in other forms of art that place value on deliberate creation. As such, any discussion on AI-generated ink paintings needs to take into account the influence of new technological developments within the broader framework of Chinese visual culture. It is likely that the reception of AI-generated paintings will also be determined by the changing institutions and learning processes within which authentic cultural expression is defined (Cao, 2026). Future studies need to address how institutions and pedagogical approaches change to accommodate more instances of AI-generated art (Liu and Wang, 2025). Furthermore, it will be essential to investigate whether the evaluative concerns held by professionals in the field lessen or increase as AI generation is normalized in the learning process (Liu et al., 2025).

More broadly, the present findings speak to the relationship among cultural authenticity, the inheritance of traditional aesthetics, and the transformation of digital media. The observed dissociation — strong perceptual-affective engagement alongside diminished cultural authenticity among expert viewers — suggests that AI-generated works may enter the cultural tradition at the level of perceptual experience before they achieve legitimacy at the level of institutional and pedagogical recognition (Cao, 2026; Liu and Wang, 2025). Whether algorithmic generation ultimately functions as a vehicle for the living transmission of ink-painting aesthetics or as a force that redefines its boundaries will depend on the institutions and learning processes through which authentic cultural expression is negotiated (Liu et al., 2025).

Notwithstanding these contributions, several limitations of the present study should be acknowledged.

First, the participant sample was drawn exclusively from university art academies and general subject pools in a Chinese and Korean cultural context, and it remains an open empirical question whether the patterns observed — particularly the positive perceptual-affective response to the stochastic visual properties of AI-generated ink works — would generalize to audiences unfamiliar with the aesthetic philosophy and visual conventions of the Chinese ink painting tradition. The cultural valorization of accident and spontaneity (‘contingency/accident’, 偶然性) that may predispose culturally informed viewers to interpret algorithmic indeterminacy positively is unlikely to operate uniformly across cross-cultural samples, and future research should explicitly recruit participants from diverse cultural backgrounds to test the boundary conditions of the expressive expansion effect.

Second, the present study did not administer a validated measure of prior attitudes toward AI-generated art prior to the experimental session. Individual differences in AI art attitudes — including perceptions of AI creativity, concerns about authenticity, and openness to algorithmic authorship — have been shown to independently predict aesthetic and authenticity judgments in prior research, and their absence as a measured covariate represents a potential confound, particularly for the cultural authenticity ratings that showed the largest between-group variance. Future studies should incorporate established AI attitude instruments at baseline to statistically control for this source of individual difference.

Third, although the three stimulus groups were matched on the six low-level visual indices examined (mean luminance, RMS contrast, edge density, color saturation, hue diversity, and spectral slope), other low-level visual properties that were not explicitly controlled — including local brushstroke density and ink diffusion texture statistics — may have differed systematically across conditions as a function of the generative process itself. These residual stimulus-level differences cannot be fully ruled out as partial contributors to the observed condition effects. More fundamentally, the AI-generated images were sampled from a large generative space and screened using computational aesthetic scores plus expert review, whereas the human-made digital works were produced by a small number of commissioned artists meeting a minimum quality threshold. This asymmetry in selection procedures means the observed differences may partly reflect unequal curation opportunity rather than an inherent property of AI generation. Future stimulus construction protocols should implement symmetric selection pipelines and incorporate more comprehensive computational matching or covariate control procedures.

Fourth, the interpretation of beta band suppression as an index of aesthetic immersion rests on an inferential chain derived from prior neuroaesthetics literature rather than from direct causal evidence in the present dataset. Beta suppression is a broadly distributed neural phenomenon with multiple functional interpretations — including motor preparation, cognitive inhibition, attentional disengagement, prediction error signaling, and task-related processing — and the present data do not permit definitive attribution of the observed beta power changes specifically to aesthetic immersion processes. Alternative neural interpretations cannot be excluded on the basis of the present evidence alone, and future studies employing source localization or convergent neuroimaging methods would provide stronger grounds for this interpretation.

Fifth, the present design employed a single observation per stimulus per participant, precluding examination of how aesthetic responses to AI-generated ink paintings evolve with repeated exposure. Aesthetic adaptation, familiarity-based preference shifts, and the potential normalization of AI authorship over time are theoretically important processes that the cross-sectional design cannot address. Longitudinal or repeated-exposure designs would be particularly valuable for investigating whether this dissociation — high expressiveness coexisting with low cultural authenticity — attenuates, stabilizes, or reverses as viewers accumulate experience with AI-generated works in this domain. Additionally, the present study treated expertise as a binary variable, yet professionals varied substantially in ink-painting training (4–15.3 years). Future research should examine expertise continuously (e.g., years of training, training intensity) and consider whether different subgroups among professionals — tradition-oriented versus innovation-oriented evaluators — show distinct response patterns.

## 6 Conclusion

The present study provides preliminary evidence that AI-generated ink paintings, under the specific experimental conditions and within the selected stimulus sets examined here, elicited a distinctive combination of higher expressive ratings, more dispersed visual exploration, and differential neural responses compared to traditional and digital non-AI works. The finding that professionals and non-professionals converged on expressiveness ratings but diverged on cultural authenticity is consistent with a dual-pathway account in which perceptual-affective and cultural-evaluative processing routes can dissociate — an interpretive framework advanced as consistent with the observed dissociation rather than a definitively established mechanism. However, the mechanisms underlying this dissociation — whether it reflects tradition-based authenticity criteria, detection of AI-like visual cues, or both — remain unresolved and require future research employing symmetric stimulus-selection procedures, source-attribution measures, and larger, more diverse artwork samples before stronger claims can be made.

From a practical perspective, two tentative implications emerge. First, the finding that expressive enhancement was observed across both expertise groups under blind viewing conditions suggests that AI-generated ink works may have potential as engaging stimuli for educational and exhibition contexts, provided that their cultural status is addressed explicitly rather than assumed. Recent research underscores that exhibition-environment factors such as lighting conditions shape emotional and neural responses in cultural exhibition halls (Zhao et al., 2025), and that the interaction between painting tones and illuminance predicts visual comfort in art galleries (Zhao et al., 2025); these environmental determinants should be considered alongside the intrinsic stimulus properties examined here when AI-generated ink works are deployed in real-world display settings. Second, the three-stage perceptual framework offers a provisional approach for evaluating how generated artworks engage with the associative and evaluative networks of a cultural tradition. Future research comparing blind and disclosed authorship conditions, employing longitudinal designs, and incorporating direct measures of perceived AI-likeness and source attribution will be necessary to determine whether the patterns observed here generalize beyond the present stimulus set and experimental paradigm.

## Data Availability

The datasets presented in this study can be found in online repositories. The names of the repository/repositories and accession number(s) can be found: Zenodo repository: https://doi.org/10.5281/zenodo.20540420.

## Appendix

Glossary of Chinese terms: For accessibility to non-Chinese-reading audiences, the following canonical Chinese ink-painting concepts used in this manuscript are explained here at first mention: (1) Ink Resonance (墨韵, mò yùn) — “ink resonance,” the tonal behavior, diffusion, and aesthetic vitality of ink wash on paper; (2) Leaving Blank (留白, liú bái) — “leaving blank,” the deliberate use of unpainted empty space as an active compositional element; (3) Breath-Rhythm / Spirit-Resonance (气韵, qì yùn) — “breath-rhythm” or “spirit-resonance,” the flowing vital energy and rhythmic movement in a work; (4) Contingency / Accident (偶然性, ǒu rán xìng) — “contingency” or “accident,” the generative role of uncontrolled, spontaneous ink behavior. These terms are retained in their original Chinese form throughout the manuscript because they are the canonical art-historical designations that lack precise single-word English equivalents, but they should be read as substantive aesthetic concepts rather than mere labels.
