# Supplementary material for: Dual-pathway processing of AI-generated Chinese ink painting: evidence from eye-tracking, EEG, and artistic expertise
Source: Front Psychol. 2026 Sep 29;17:1966943. doi: 10.3389/fpsyg.2026.1966943 (PMC13658077; doi:10.3389/fpsyg.2026.1966943)
Supplement: Supplementary file 1 [file Supplementary_File_1.docx]

# Supplementary Materials

Dual-Pathway Processing of AI-Generated Chinese Ink Painting: Evidence from Eye-Tracking, EEG, and Artistic Expertise

Qiang Qin, Jing Chai, Wanni Zhong*
Frontiers in Psychology — Manuscript ID 1966943
Corresponding author: Wanni Zhong (zwn97z@163.com)

## Supplementary Table S1. Semantic differential instrument — the 20 bipolar adjective pairs

**Supplementary Table S1.** The 20 bipolar adjective pairs of the semantic differential instrument, organized by subscale. Each item was rated on a 0–100 continuous scale (visual-analog slider with integer steps); subscale scores were computed as the mean of the constituent items.

| **Subscale** | **Bipolar adjective pair** |
| --- | --- |
| Expressiveness | dynamic–static; energetic–calm; powerful–delicate; vivid–muted; expressive–inexpressive |
| Authenticity | authentic–inauthentic; genuinely ink-painting–not genuine; rooted in tradition–detached from tradition; artistically sincere–artificial; culturally legitimate–illegitimate |
| Cultural resonance | culturally resonant–culturally neutral; evocative of ink tradition–not evocative; connected to Chinese aesthetic heritage–disconnected; meaningful in cultural terms–meaningless; tradition-associated–tradition-independent |
| Innovativeness | innovative–conventional; novel–familiar; original–derivative; pioneering–traditional; unprecedented–commonplace |

Note. The four-factor structure was confirmed by confirmatory factor analysis (maximum likelihood, lavaan 0.6-12): χ²(164) = 218.43, p = .004; CFI = 0.96; TLI = 0.95; RMSEA = 0.058 (90% CI [0.031, 0.079]); SRMR = 0.061; factor loadings 0.61–0.84 (all p < .001); inter-factor correlations 0.21–0.47; internal consistency α > 0.80 for all subscales.

## Supplementary Table S2. Low-level visual matching across stimulus conditions

**Supplementary Table S2.** One-way ANOVA results for the six low-level visual indices computed for each of the 60 stimuli (20 per condition). The three stimulus conditions did not differ significantly on any index.

| **Index** | **F(2, 57)** | **p** | **η²p** |
| --- | --- | --- | --- |
| Mean luminance (CIE L*) | 1.43 | .248 | .048 |
| RMS contrast | 1.87 | .163 | .062 |
| Edge density (Canny) | 2.11 | .131 | .069 |
| Color saturation (HSV) | — | > .05 | — |
| Hue diversity (HSV SD) | — | > .05 | — |
| Spectral slope (1/f) | — | > .05 | — |

Note. The three primary indices (mean luminance, RMS contrast, edge density) are reported in Section 3.2 of the main text. Color saturation, hue diversity, and spectral slope likewise showed no significant between-group differences (all p > .05); their complete statistics are available in the study's reproducibility package.

## Supplementary Note. R environment specification

All statistical analyses were conducted in R version 4.4.0 using the following packages: ez (4.4-0), emmeans, lme4, lmerTest, effectsize, and lavaan (0.6-12).
